# Sand Flies and Pathogens in the Lowlands of Emilia-Romagna (Northern Italy)

**DOI:** 10.3390/v14102209

**Published:** 2022-10-07

**Authors:** Mattia Calzolari, Giuseppe Romeo, Martina Munari, Paolo Bonilauri, Roberta Taddei, Maria Sampieri, Simone Bariselli, Gianluca Rugna, Michele Dottori

**Affiliations:** Istituto Zooprofilattico Sperimentale della Lombardia e dell’Emilia Romagna, Via Bianchi 9, 25124 Brescia, Italy

**Keywords:** *Phlebovirus*, Toscana virus, Fermo virus, *Leishmania infantum*, *Phlebotomus perfiliewi*, *Phlebotomus perniciosus*

## Abstract

Cases of sand fly-borne diseases in the Emilia-Romagna region, such as meningitis caused by Toscana virus and human leishmaniasis, are reported annually through dedicated surveillance systems. Sand flies are abundant in the hilly part of the region, while the lowland is unsuitable habitat for sand flies, which are found in lower numbers in this environment with respect to the hilly areas. In this study, we retrieved sand flies collected during entomological surveillance of the West Nile virus (from 2018 to 2021) to assess their abundance and screen them for the presence of pathogens. Over the four-year period, we collected 3022 sand flies, more than half in 2021. The most abundant sand fly species was *Phlebotomus (Ph.) perfiliewi*, followed by *Ph. perniciosus*; while more rarely sampled species were *Ph. papatasi*, *Ph. mascittii* and *Sergentomyia minuta*. Sand flies were collected from the end of May to the end of September. The pattern of distribution of the species is characterized by an abundant number of *Ph. perfiliewi* in the eastern part of the region, which then falls to almost none in the western part of the region, while *Ph. perniciosus* seems more uniformly distributed throughout. We tested more than 1500 female sand flies in 54 pools to detect phleboviruses and *Leishmania* species using different PCR protocols. Toscana virus and *Leishmania infantum*, both human pathogens, were detected in 5 pools and 7 pools, respectively. We also detected Fermo virus, a phlebovirus uncharacterized in terms of relevance to public health, in 4 pools. We recorded different sand fly abundance in different seasons in Emilia-Romagna. During the season more favorable for sand flies, we also detected pathogens transmitted by these insects. This finding implies a health risk linked to sand fly-borne pathogens in the surveyed area in lowland, despite being considered a less suitable habitat for sand flies with respect to the hilly areas.

## 1. Introduction

Phlebotomine sand flies are small hematophagous insects, which can be a great nuisance to humans in the most suitable environments for these insects, because they can reach significant population densities [1]. In addition to the nuisance, sand flies can be a significant public health problem, since they act as vectors of multiple diseases caused by different pathogens (from parasites to bacteria and viruses) [2,3]. These insects are widespread in Mediterranean countries and abundant in large parts of Italy, with their ideal habitat in hills and coastal plains [4,5]. In the Emilia-Romagna region, these insects find their most suitable environment in the northern foothills bordering the Apennine Mountains. This area hosts a large population of sand flies, in particular the species *Ph. perfiliewi*, which can be sampled in thousands of specimens using attractive traps [6]. In contrast, the region’s lowlands are a less favorable environment, in which sand flies are considered scarce or absent [5,7,8,9]. The abundance of these insects varies greatly within each season and from season to season [6]. They have a patchy distribution and sudden peaks in density, which hampers their sampling [10,11].

Human cases of meningitis caused by Toscana virus (TOSV), a virus of the *Phlebovirus* genus, are reported annually in Emilia-Romagna [12]. In addition, several other phleboviruses have been reported, while their relevance to public health is so far uncharacterized [13,14]. Interestingly one of these viruses, the Fermo virus (FERV), has been detected using serological methods in domestic animals, mainly in goats, suggesting a possible involvement of these animals in its cycle [15].

A re-emergence of human visceral and cutaneous leishmaniasis has been reported in Emilia-Romagna in the last decade [16,17,18,19]. The low prevalence of canine leishmaniasis in the Region—as compared to other endemic areas in Italy—and molecular data on strains of *Leishmania (L.) infantum* suggest a different epidemiological cycle of the parasite in Emilia- Romagna, which likely does not involve dogs as principal reservoirs [20].

Sand fly-borne diseases are actively monitored in Emilia-Romagna together with arboviruses transmitted by mosquitoes. Intensive entomological surveillance was established for monitoring West Nile and Usutu viruses in the plain of the region [21]. We used sand flies collected during this surveillance to evaluate their abundance, to assess the infection of sand flies and the pathogens they transmit in the lowlands of Emilia-Romagna.

## 2. Material and Methods

### 2.1. Survey Area

The Emilia-Romagna region covers around 22.500 km^2^ and has a human population of 4.5 million. It has a nearly triangular shape, elongated for 230 km from west to east, where it reaches the sea with a coastline stretching for 135 km.

**Figure 1:**
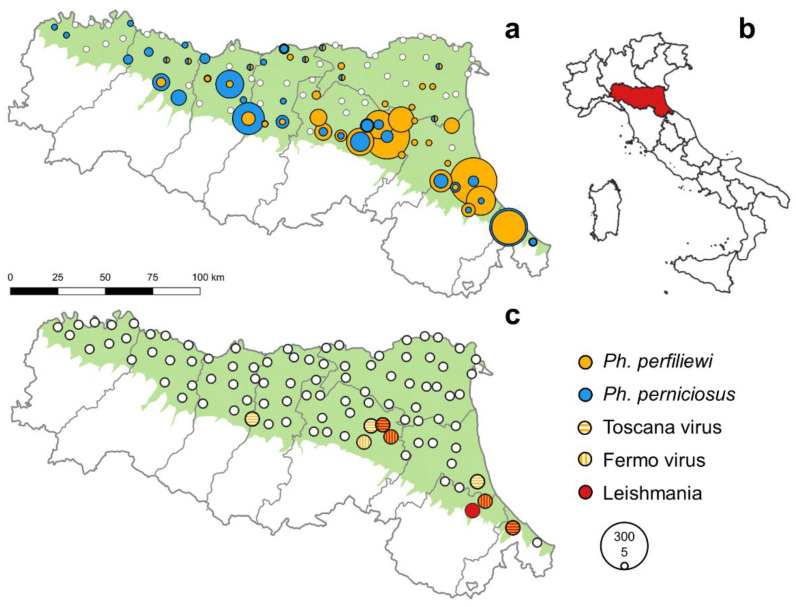
Number of *Ph. perfiliewi* and *Ph. perniciosus* collected in 2021 (number proportional to circle size) at the different sites on the survey area (green) (**a**) with reference to the location of Emilia-Romagna Region on the map of Italy (**b**) and pools positive for tested pathogens (**c**).

Part of the principal plain in Italy, the Po Valley, is located in the northern part of the region, while the northern border of the Apennines stretches to the south, and there is a hilly area located between the two (Figure 1). The survey covered the plain area of the region, which represents about a half of its surface, characterized by high human population density and intensive agricultural activity. The climate is sub-continental, with hot and humid summers and cold winters, and sub-Mediterranean along the coast. The average temperature is 12.7 °C (1981–2010), the average minimum and maximum temperatures are 7.7 °C and 17.7 °C, respectively; annual precipitation is between 650 and 1200 mm [21]. In the period 1991–2015, compared with 1961–1990, the average temperatures increased by 1.1 °C (+1.4 °C maximum and +0.8 °C minimum temperatures), while annual precipitation fell by only 22 mm (−2%), but with significant seasonal changes (with a trend to drier summers and wetter falls) [22].

The accumulated precipitation between May and October in the different years of sampling was determined by retrieving daily data from 28 weather stations in the plain area of Emilia-Romagna (Figure S1). The average of daily data was used to obtain the accumulated precipitation. Data was obtained by the DEXT3R on-line service (https://simc.arpae.it/dext3r/ accessed on 10 April 2022) managed by ARPAE (regional environment agency).

### 2.2. Sand Fly Sampling and Identification

The sand flies used in this study were collected as part of the entomological plan for WNV surveillance, from 2018 to 2021. The plan involves collecting samples in a network of 95 fixed sites, distributed throughout the plain in Emilia-Romagna, according to a grid of 11 km (Figure 1). At every site a CO_2_ trap, working overnight, was activated once every two weeks, acquiring the same number of samplings for every site in the same season. Trap sites were georeferenced and showed an altitude ranging from −4 m to 122 m, with an average of 22.7 m. The starting day of surveillance was brought forward over the seasons, from June 12 in 2018 to the beginning of May in 2020 and 2021, and ended at the beginning of October. More details on the plan have been reported elsewhere [21].

We killed live insects by posing the collection bag at −20 °C for about 15 min, then we retrieved sand flies, separating males and females. The males were clarified in chlorine-lacto-phenol and morphologically identified under a light microscope using specific morphological keys [23,24], if the number of females collected was less than 10, also females were morphologically identified. The females in samples with 10 females or more were grouped in pools (with a maximum of 100 insects per pool), and conserved at –80 °C until the molecular testing.

### 2.3. Detection of Pathogens

The pools of sand flies were manually ground in PBS, using pellet pestle, then genetic material was extracted using an automated extractor (BioSprint 96, Qiagen, Hilden, Germany). A specific real-time protocol was used to detect the kinetoplast DNA of *L. infantum* parasites [25]. The extracted RNA was retro-transcribed and phlebovirus detection was performed using PCR (hereinafter pan-phlebovirus PCR), targeting a 370-nucleotide region of the S segment [26]. The amplicons obtained through the pan-phlebovirus PCR were sequenced and deposed in GenBank (AN: OP485761, OP485762, OP485763, OP485764). The sequences were used to identify viruses detected by BLAST analysis in the GenBank database (https://blast.ncbi.nlm.nih.gov/Blast.cgi accessed on 10 April 2022). The TOSV was searched for using a specific real-time PCR [27]. The minimum infection rates (MIR) of *Leishmania*, TOSV and FERV were calculated for every sample, assuming one positive sand fly per positive pool.

Pathogen and sand fly distribution maps were produced using QGIS software 3.4 (QGIS.org, 2022. A Free and Open Source Geographic Information System. QGIS Association).

## 3. Results

A total of 3,022 sand flies were collected in the four years of sampling. The number of sand flies collected annually from 2018 to 2020 ranged from 99 to 405, while the highest number of sand flies (2400 specimens) was collected in 2021 (Table 1). The number of sand flies sampled in May was negligible (only two in 2020) (Table S1). We subjected 1480 sandflies to morphological identification after chlorine-lacto-phenol clarification, the most represented species was *Ph. perfiliewi* (53% of identified sand flies), followed by *Ph. perniciosus* (46.4% of identified sand flies), which was the most sampled species only in 2020. Few specimens of *Ph. papatasi, Ph. mascittii* and *Sergentomyia (Se.) minuta* were collected (less than 0.3% each; Table 1). The geographical pattern of the species distribution does not differ from season to season, with a higher number of *Ph. perfiliewi* in the eastern part of the region, falling to almost none in the west. *Ph. perniciosus* showed a more homogeneous distribution, with comparable abundance rates throughout the southern part of the survey area (Figure 1).

The high number of sand flies collected in 2021, allowed us to test the insects collected during this year: 1542 female sand flies were grouped in 54 pools, with an average of 29 specimens per pool. Five pools tested positive for TOSV and we detected sequences ascribable to another phlebovirus in four other pools. These sequences originated from FERV (GenBank AN: OP485761, OP485762, OP485763, OP485764), as shown by the high identity (>98.5%) obtained by deposed sequences of the same virus. *Leishmania* parasites were detected in seven pools (Table 2). One pool tested positive for both *L. infantum* and TOSV, while we detected TOSV or FERV and *L. infantum* on different sampling days at three sites (Table 3): therefore, we detected both *L. infantum* and TOSV or FERV at four sites (Figure 1). The positive pools were all detected in the eastern part of the region at sites where more sand flies were collected and where *Ph. perfiliewi* was more abundant (Figure 1).

**Figure 2:**
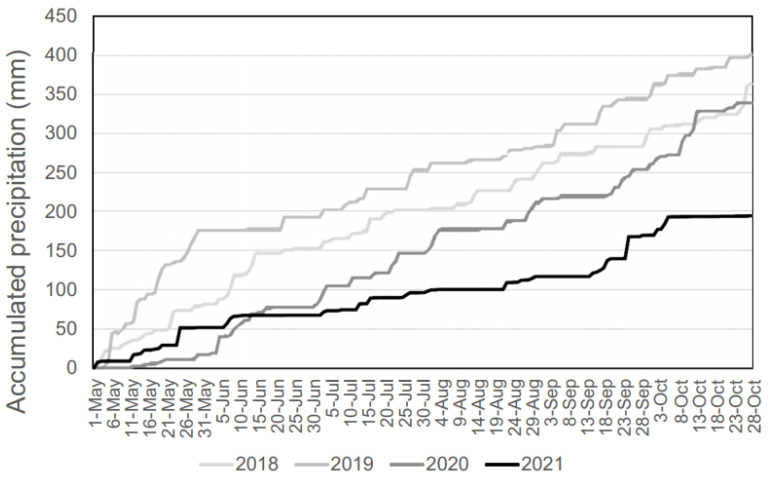
Accumulated precipitation from May to October in the four years of survey in the plain of Emilia-Romagna.

The accumulated precipitation in the plain area in the different seasons point out the 2021 as the drier season between years of sampling, with 193 mm against values ranging from 339 and 401 in the other seasons (Figure 2).

## 4. Discussion

High rates of sand fly abundance had already been recorded in Emilia-Romagna in early studies conducted from the 1960s, which recorded mostly *Ph. perfiliewi* and a low prevalence (less than 10%) of *Ph. perniciosus* [6,7,8] or *Ph. perfiliewi* alone [4,7]. More recent entomological monitoring confirmed this situation in terms of abundance and species composition, with an overwhelming presence of *Ph. perfiliewi* [9,10,11,12].

All these studies recognized the hilly environment in the central and eastern part of the region as the most suitable to find abundant populations of sand flies. Lowland areas are known to be less suitable for the sand flies’ presence, which are considered scarce or absent due to less favorable climatic and environmental conditions, perhaps also as a consequence of the use of agrochemicals and pesticides on crops [8].

Our study, which includes samples from different seasons, revealed the presence of sand flies in the lowland of the region, although with total numbers fewer than those recorded in the hills. We found a great difference in abundance of sand flies between the different sites and areas of the lowland, with some surveyed sites without collected sand flies. The species in the plain are the same as those found in the hills, and their abundance, while lower, reflects a similar trend in geographical distribution, with a decrease of *Ph. perfiliewi* abundance from east to west. The relative proportion between more abundant species in the lowland is different from the hills, as *Ph. pernicious* represents about a half of identified sand flies in the plain, while is less than 10% in the hills.

The low number of individuals belonging to species other than *Ph. perniciosus* and *Ph. perfiliewi* is possibly linked to the location of sampling sites—rural and semi-natural environments—while *Ph. papatasi*, in our experience, is more common in environments affected by human activity, such as farms and old houses. The scarce presence of *Ph. mascittii* is linked to its actual rarity; few specimens have been recorded in Emilia-Romagna so far [28]. The low number of the sand fly *Se. minuta* is partially due to the trap used, which is less attractive for this species [29]. *Se. minuta* seems less epidemiologically relevant, as this sand fly feeds preferentially, but not exclusively, on cold-blooded animals [30].

The data obtained revealed a strong variability in the number of sand flies collected in different seasons. A remarkable difference in population densities in different seasons had already been recorded in the hills of Emilia-Romagna, where a more abundant sand fly population was linked to lower precipitation rates in spring and early summer [9]. Notably, the year with the highest prevalence of sand flies (2021) featured a drier spring/summer period compared to other surveillance seasons, as pointed out by accumulated precipitation. This observation seems to confirm that such dry conditions are an enhancing factor in sand fly abundance in Emilia-Romagna, maybe increasing the suitability of ecological niche of sand fly, particularly of larval habitats. This suggests that sand flies are finding increasingly suitable conditions, given the trend in dry summers observed in recent years in the region.

In 2021, the high number of sand flies collected in the plain was sufficient for performing molecular tests for pathogens already detected in Emilia-Romagna, particularly *L. infantum* and TOSV. Moreover, the application of the pan-phlebovirus PCR allowed the detection of FERV, although this type of protocol can underestimate the presence of a virus compared to a specific PCR. Interestingly, positive pools were recorded in the southwestern part of the survey area, where the rate of sand fly abundance is high and cases of disease in humans have mainly been reported. The positive pools were collected in the sites where the most sand flies were collected. This data seems to confirm a possible relationship between the abundance of sand flies and the circulation of the pathogens they transmit, as already hypothesized for TOSV on a serological basis [31] and already observed in sand flies collected at one site for *L. infantum* and phleboviruses [9]. In this case, the relationship between dry season and abundance of sand flies would also involve a higher circulation of pathogens, an association already made in a study of a leishmaniasis outbreak recorded in Emilia-Romagna in 1970 [7,32].

Finally, with regard to the detection of FERV, even though the pathogenicity of this virus is still uncharacterized, its circulation in the plain area of Emilia-Romagna calls for a better characterization of its cycle. Moreover, a clear definition of the potential pathogenicity of this virus is needed, in order to characterize its relevance to human and veterinary health.

This study demonstrates the added value of an entomological surveillance plan adopting regular sampling in terms of geographical area and time, and an intensive sampling effort, which can provide data on untargeted pathogens. Surveillance projects in place to monitor the spread of arboviruses and leishmaniasis have reported human cases of leishmaniasis and TOSV meningitis mainly in people living in the hilly parts of the region, but also, to a lesser extent, in people living in the plain area. The results obtained in this study confirm that, in favorable seasons, infections can also occur in the lowland environment, constituting a real risk to human health. Since control of these insects is difficult due to the impossibility of locating and reaching larval breeding sites, protection against sand fly bites —use of protective clothing to minimize skin exposure, application of adequate insect repellent (e.g., containing DEET), use of fine-mesh nets, use of residual insecticide in the home—should be adopted in these areas, as the most effective strategy against infection [1,10,11].

## Figures and Tables

**Table 1:** Sand flies collected in the different years of sampling by specimens identified.

	2021	2020	2019	2018	Total
*Phlebotomus perfiliewi*	484	170	72	58	784
*Phlebotomus perniciosus*	370	232	45	40	687
*Phlebotomus mascittii*	2	2			4
*Sergentomyia minuta*	2		1	1	4
*Phlebotomus papatasi*		1			1
*Phlebotomus* spp. *	1542				1542
Total	2400	405	118	99	3022

* Subjected to molecular testing.

**Table 2:** Sand flies collected and tested in 2021 by the number of pools tested and the pathogens detected.

Province	Sampled	Tested	Pools	TOSV	FERV	*Leish.*
Bologna	765	515	16	2	3	3
Forlì Cesena	247	188	7		1	3
Ferrara	154	105	5			
Modena	233	121	6	1		
Piacenza	20					
Parma	104	46	4			
Ravenna	342	288	6	1		
Reggio Emilia	150	93	4			
Rimini	385	186	6	1		1
Total	2400	1542	54	5	4	7

**Table 3:** Pools that tested positive to the different pathogens, with reference to the minimum infection rate (MIR), by province and municipality and day of sampling.

						Pool tested positive (MIR)	
Province	Municipality	Site code	Day of sampling	Tested (N/Pools)	TOSV	FERV	*Leish*.
Bologna	Budrio	WN109A	29/06/2021	57/1		1(1.8)	
		WN109A	10/08/2021	24/1		1(4.2)	
		WN096A	20/07/2021	16/1	1(6.3)		
	Medicina	WN110A	06/07/2021	25/1		1(4.0)	
		WN110A	20/07/2021	165/2			1(0.6)
		WN110A	14/09/2021	36/1			1(2.8)
	Molinella	WN097B	22/06/2021	10/1	1(10)		
		WN097B	14/09/2021	23/1			1(4.3)
Forlì Cesena	Cesena	WN133B	19/08/2021	20/1		1(5.0)	
		WN133B	02/09/2021	38/1			1(2.6)
		WN133B	16/09/2021	31/1			1(3.2)
		WN136A	16/09/2021	10/1			1(10)
Modena	Formigine	WN090A	20/07/2021	12/1	1(8.3)		
Ravenna	Ravenna	WN129B	15/07/2021	192/2	1(0.5)		
Rimini	Rimini	WN138A	16/09/2021	27/1	1(3.7)		1(3.7)

## Data Availability

Part of the data presented in this study are openly available in GenBank (https://blast.ncbi.nlm.nih.gov/Blast.cgi accessed on 10 April 2022). Publicly available datasets were analyzed in this study. This data can be found here: https://simc.arpae.it/dext3r/ accessed on 10 April 2022.

## Supplementary Materials

The following supporting information can be downloaded at: https://www.mdpi.com/article/10.3390/v14102209/s1, Figure S1: Location on the Emilia-Romagna map of the weather stations used for calculation of the accumulated precipitation; Table S1: Sampled sand flies with reference to the year and month of sampling.
