# Supplementary material for: Sand Flies and Pathogens in the Lowlands of Emilia-Romagna (Northern Italy)
Source: Viruses. 2022 Oct 7;14(10):2209. doi: 10.3390/v14102209 (PMC9608450; doi:10.3390/v14102209)
Supplement: Supplementary file 1 [file viruses-14-02209-s001.zip › viruses-1888882-supplementary.pdf]

Supplementary Material

Table S1. Sampled sand flies with reference to the year and month of sampling.

	Year	May	June	July	August	Sept.	Total
Phlebotomus perfiliewi	2018			25	22	11	58
	2019		1	30	35	6	72
	2020	1	5	135	29		170
	2021		22	141	182	139	484
Phlebotomus perniciosus	2018			15	19	6	40
	2019		8	27	10		45
	2020	1	3	140	55	33	232
	2021		33	178	99	60	370
Phlebotomus papatasi	2020			1			1
Phlebotomus mascittii	2020		1	1			2
	2021		1	1			2
Sergentomyia minuta	2018			1			1
	2019			1			1
	2021				2		2
Phlebotomus sp.	2021		77	929	308	228	1542
		2	151	1625	761	483	3022

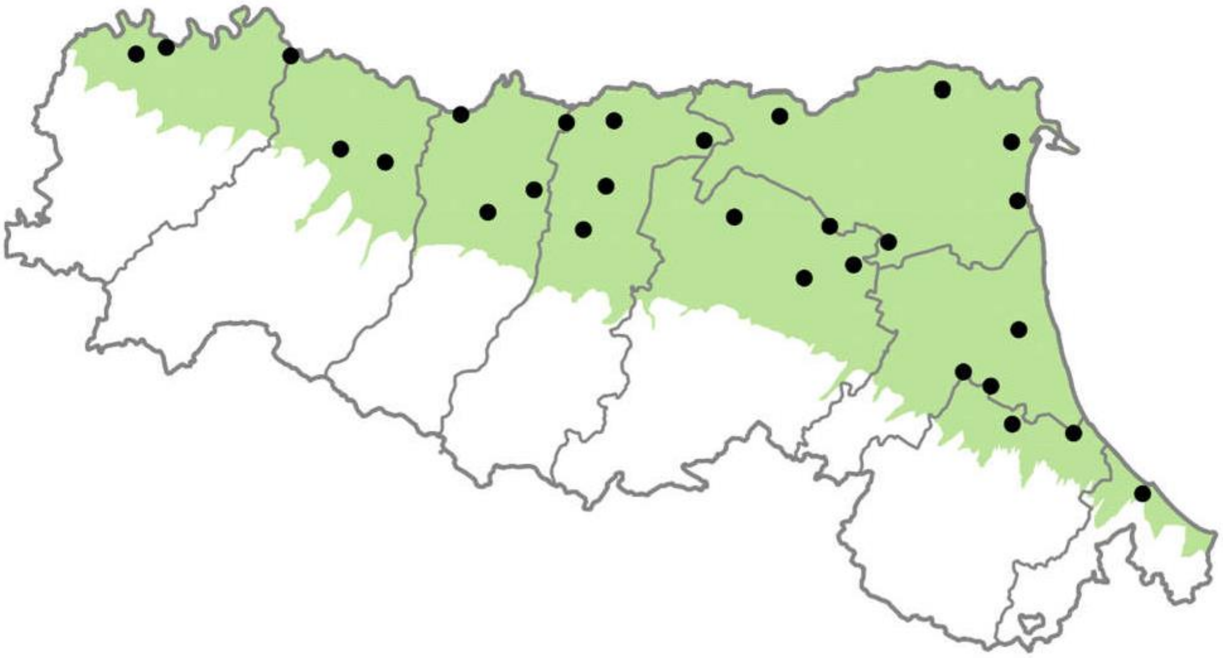

Figure S1. Location on the Emilia-Romagna map of the weather stations used for calculation of the accumulated precipitation.
